# *Kalrn *promoter usage and isoform expression respond to chronic cocaine exposure

**DOI:** 10.1186/1471-2202-12-20

**Published:** 2011-02-17

**Authors:** Richard E Mains, Drew D Kiraly, Jodi E Eipper-Mains, Xin-Ming Ma, Betty A Eipper

**Affiliations:** 1 Department of Neuroscience University of Connecticut Health Center 263 Farmington Ave Farmington CT 06030-3401 USA; 2 Department of Genetics and Developmental Biology University of Connecticut Health Center 263 Farmington Ave Farmington CT 06030-3401 USA

## Abstract

**Background:**

The long-term effects of cocaine on behavior are accompanied by structural changes in excitatory glutamatergic synapses onto the medium spiny neurons of the striatum. The *Kalrn *gene encodes several functionally distinct isoforms; these multidomain guanine nucleotide exchange factors (GEFs) contain additional domains known to interact with phosphatidylinositides as well as with a number of different proteins. Through their activation of Rho proteins and their interactions with other proteins, the different Kalirin isoforms affect cytoskeletal organization. Chronic exposure of adult male rodents to cocaine increases levels of Kalirin 7 in the striatum. When exposed chronically to cocaine, mice lacking Kalirin 7, the major adult isoform, fail to show an increase in dendritic spine density in the nucleus accumbens, show diminished place preference for cocaine, and exhibit increased locomotor activity in response to cocaine.

**Results:**

The use of alternate promoters and 3'-terminal exons of the mouse *Kalrn *gene were investigated using real-time quantitative polymerase chain reaction. While the two most distal full-length *Kalrn *promoters are used equally in the prefrontal cortex, the more proximal of these promoters accounts for most of the transcripts expressed in the nucleus accumbens. The 3'-terminal exon unique to the Kalirin 7 isoform accounts for a greater percentage of the *Kalrn *transcripts in prefrontal cortex than in nucleus accumbens. Western blot analyses confirmed these differences. Chronic cocaine treatment increases usage of the promoter encoding the Δ-Kalirin isoforms but does not alter full-length Kalirin promoter usage. Usage of the 3'-terminal exon unique to Kalirin 7 increases following chronic cocaine exposure.

**Conclusions:**

*Kalrn *promoter and 3'-terminal exon utilization are region-specific. In the nucleus accumbens, cocaine-mediated alterations in promoter usage and 3'-terminal exon usage favor expression of Kalirin 7 and Δ-Kalirin 7. The Δ-isoform, which lacks a Sec14p domain and four of the nine spectrin-like repeats found in full-length Kalirin isoforms, increases spine headsize without increasing dendritic spine numbers. Thus cocaine-mediated changes in alternative splicing of the *Kalrn *gene may contribute importantly to the behavioral, morphological and biochemical responses observed.

## Background

Despite the expression of multiple Rho GDP/GTP exchange factors (GEFs) in the nervous system, the *Kalrn *gene, which encodes multiple isoforms of Kalirin, plays an essential, non-redundant role. Linkage analyses identified *Kalrn *as a major risk factor for coronary artery disease and stroke [1-3], as well as for schizophrenia and adult attention-deficit/hyperactivity disorder [4-6]. In addition, Kalirin interacts with many different proteins including deleted in schizophrenia 1 (DISC1), a major risk factor for schizophrenia, the inducible form of nitric oxide synthase (iNOS; NOS2), Huntingtin-associated protein 1 (HAP1) and peptidylglycine α-amidating monooxygenase, an essential enzyme for neuropeptide biosynthesis [7-11]. Chronic cocaine treatment increases levels of Kal7 protein in the mouse and rat striatum [12]. Estrogen treatment of ovariectomized female rats increases Kalirin expression [13], and Alzheimer's Disease is accompanied by a dramatic loss of Kal7 in the hippocampus [14]. Hippocampal Kalirin mRNA is elevated at 4 h following a kainate-induced seizure and remains elevated for a month [15].

Kalirin has been implicated in many functions. Kalirin is one of a small number of RhoGEFs localized to the postsynaptic density (PSD) [16]. In the adult brain, Kalirin 7 (Kal7) is the predominant isoform and is almost exclusively localized to the PSD [17,18]. Overexpression of Kal7 leads to increases in dendritic spine number in cultured hippocampal and cortical pyramidal neurons [19-21]. Conversely, a specific reduction of Kal7 in primary neurons causes a dramatic decrease in spine density [13,21]. Studies on Kalirin have focused on cortex and hippocampus, where Kal7 has a clear role in spine formation and maintenance.

With many functional domains, the different Kalirin isoforms are capable of integrating inputs from a variety of surface molecules (ephrins, N-cadherin, Trk receptors), phosphatidylinositides, cytoskeletal components, PDZ domain proteins like spinophilin and PSD-95, plus signaling molecules and growth factors [18,22-25]. Kal7 is also crucial for the expression of normal long-term potentiation in CA1 hippocampal pyramidal neurons [17].

The rat *Kalrn *gene encompasses more than 65 exons spanning 600 kb of the rat genome, with at least six promoters and five 3'-terminal exons [26-28]. Alternative splicing yields multiple functionally different isoforms of rat Kalirin. We recently developed a mutant mouse specifically lacking the Kal7 isoform (Kal7^KO^) [17]; the 3'-terminal exon that encodes a PDZ binding motif known to interact with PSD-95 and AF-6 was eliminated [18,25]. Kal7^KO ^mice show decreased anxiety-like behavior, impaired acquisition of a passive avoidance task, and abnormal behavioral and morphological responses to chronic cocaine [12,17]. A total Kalirin knockout mouse (T-Kal^KO^) mice, in which exons 27-28 in the first GEF domain were replaced by a neomycin resistance gene, was also developed [29]. T-Kal^KO ^mice also display a number of neurological phenotypes. Use of these mouse models to clarify the role of Kalirin proteins in synaptic plasticity requires better characterization of the major products of the mouse *Kalrn *gene.

Given the essential role of Kal7 in multiple cocaine-elicited behaviors as well as the interactions of Kalirin with cyclin dependent kinase 5 (Cdk5) and the NR2B subunit of the NMDA receptor [12,17,18,30], proteins important for these behaviors, we chose to examine more closely the effects of cocaine on the expression of different Kalirin isoforms in the striatum and, more specifically, in the ventral striatum or nucleus accumbens. In order to identify the striatal cell types expressing Kal7, we took advantage of mice expressing GFP under control of the D1-dopamine receptor promoter.

## Methods

### Cocaine treatment of mice

Mice were kept in the University of Connecticut Health Center animal facility on a 12 h light/dark cycle (lights on 7:00 am to 7:00 pm) and handled in accordance with University of Connecticut Health Center Institutional Animal Care and Use Committee guidelines. Adult male C57BL/6 mice (Jackson Laboratories; 2-5 months old) were treated daily with cocaine (20 mg/kg/day, i.p.) or saline for 7 days [12] and sacrificed 24 h after the final injection. When tested, the mice exhibited the expected locomotor sensitization to chronic cocaine administration (not shown) [12]. Mice expressing green fluorescent protein (GFP) under the control of the D1-dopamine receptor promoter were from the University of Missouri Mutant Mouse Regional Resource Center (Tg(Drd1a-EGFP)X60Gsat/Mmmh). These mice were bred at least seven generations into C57Bl/6 (Jackson Labs) before use.

### Quantitative polymerase chain reaction (Q-PCR)

RNA was prepared from freshly dissected tissues with Trizol (Invitrogen) following the manufacturer's instructions, except that the isopropanol precipitation step was lengthened to overnight at -20°C and there were 2 ethanol washes instead of one. The cDNA samples were prepared using Superscript II (Invitrogen) and random primers, or iScript (BioRad) with random primers; a 5 min 65°C step was added to the iScript protocol before chilling and adding the enzyme. While GAPDH and actin samples were unaltered by the choice of random priming or oligo-dT priming, Kalirin transcripts, which are much longer, appeared to be 10-50-fold less abundant compared to GAPDH when using oligo-dT priming instead of random priming. Pairwise statistical comparisons used the t-test for two samples assuming unequal variance and the two-tailed value is reported.

Real-time PCR was performed using an Eppendorf Realplex2 machine and Sybr-Green (BioRad), with the default parameters (95°C 2 min; 95°C 15 sec, 55°C 15 sec, 68°C 20 sec; repeat 40x) except that the 68°C elongation was lengthened to 40 sec. Maximal rates of amplification per cycle were calculated for all primer pairs and all samples in all assays and averaged 1.98 ± 0.06 (SD) for a set of 8 consecutive 96-well assays. Data are calculated with respect to GAPDH for each sample within an assay; since values with respect to GAPDH for each transcript were consistent across assays, data were averaged. Use of actin instead of GAPDH for normalization did not alter the results. Primers are listed in Table 1.

**Table 1:** Primers used for quantitative polymerase chain reaction

Gene Name	Oligo Name	Sequence	T_m _(°C)	Length (nt)
GAPDH	mrGapdh-top	TTGTCAGCAATGCATCCTGCACCACC	61	119
	mrGapdh-rev2bot	CTGAGTGGCAGTGATGGCATGGAC	61	

**3'-terminal exons**				

Kalirin7-specific	KalEx33-for	GATACCATATCCATTGCCTCCAGGACC	61	127
	Kal7unique-rev	CCAGGCTGCGCGCTAAACGTAAG	62	

Kalirin8-specific	KalEx38-for	CTGACGTGCCTACTGCTGCAGAC	61	130
	Kal8unique-rev	CTGGTTGAGGTCCTGGGAGCCAC	62	

Kalirin9-specific	KalEx51-for	GCCCCTCGCCAAAGCCACAGC	62	125
	Kal9unique-rev	CCAGTGAGTCCCGTGGTGGGC	62	

Kalirin12-specific	KalEx62-for	CAGCAGCCACGTGCCTGCAGC	62	140
	KalEx62-rev	TCTTGACATTGGGAATGGGCCGCAC	61	

**Promoter-specific**				

Kalirin A promoter	KalPromA-for	CAGGGCAGCCATAAATGGTTTTATCTG	58	121
	KalEx3-rev	CCTCGCTTGTCACGCCCCC	60	

Kalirin B promoter	KalPromB-for	GTGGACGCCTTTTTCCGGACAG	59	116
	KalEx3-rev	CCTCGCTTGTCACGCCCCC	60	

Kalirin C promoter	KalPromC-for	CTTGCTTCGGCTTCTGGATCGAG	59	117
	KalEx3-rev	CCTCGCTTGTCACGCCCCC	60	

Kalirin D promoter	KalPromD-for	CTTTGTTTCTTCCTCACTGCAGCGG	59	120
	KalEx3-rev	CCTCGCTTGTCACGCCCCC	60	

ΔKalirin promoter	ΔKalProm-for	ATCCCAGGGTGAGCAGTGAGAAGG	61	144
	mKal-Ex11-rev	ATTCCCCAGTCTGAGCCAGCTGC	61	

Full-length Kalirins	mKal-Ex10-for	GCCTTTCTCAGCAAACACACTGGGG	61	157
	mKal-Ex11-rev	ATTCCCCAGTCTGAGCCAGCTGC	61	

All primer pairs were chosen to keep the melt temperature (T_m_) between 58 and 62°C (calculated using http://www.basic.northwestern.edu/biotools/oligocalc.html) and products in the 135 ± 20 nt size range. Primers were designed to delineate the unique 5' end of ΔKalirins (AF229255.1), the spectrin domains common to all forms of Kalirin with nine spectrin-like repeats (U88157.1), and sequences unique to the 3' ends of Kal7 (AF230644.1), Kal8 (U88157.1), Kal9 (AF232668.1), and Kal12 (NM_032062.2); rat sequences were used to identify the corresponding mouse sequences, which all appear as Ensembl sequences at http://www.ensembl.org/Mus_musculus/Info/. Additional primer pairs identifying transcripts which extend beyond a unique end (e.g. Exon 33 to Exon 34, Exon 38-39, Exon 51-52) instead of terminating in a specific 3' end, corroborated the major findings (not shown).

Putative mouse *Kalrn *mRNA initiation exons A, B, C and D were identified by homology to the rat and human *Kalrn *initiation exons [27,28], as described here. Mouse *Kalrn *exons A, B, and C were identified by comparing the known rat sequences (A: Genbank U88157.1; B: U88156.1; C: AF230644.1) with the mouse genome (July 2007 [NCBI37/mm9]) at http://genome.ucsc.edu/cgi-bin/. The putative mouse *Kalrn *D initiation exon sequence was from XM_001001454.1, and the putative mouse Δ*Kalrn *initiation exon sequence was from ENSMUST00000114949. The A, B, and C sequences all fit the current Ensembl sequences, and C is also a Refseq entry: NM_177357.

### Immunofluorescence analysis

Adult male Drd1a-GFP mice were anesthetized with ketamine/xylazine and perfused transcardially with saline followed by 4% paraformaldehyde as described [13]. Brains from two mice were postfixed in 4% paraformaldehyde and then incubated in 25% sucrose in phosphate buffered saline containing 0.02% NaN_3_. Coronal sections (12 μm) through the striatum and nucleus accumbens were collected on slides and incubated with rat monoclonal antibody to GFP (Nacalai USA, Elkton MD) and affinity-purified rabbit polyclonal antibody to the C-terminus of Kal7 [17,18]. Anatomical boundaries were determined as in our previous studies using standard atlases [31,32]. Confocal images were acquired on a Zeiss LSM 510 microscope as described [13,21]. Neurons expressing GFP and/or Kal7 were identified using MetaMorph; three sections, each of which contained ~40 neurons, were analyzed from each animal for each brain region. Since the minimum distance between sections analyzed using the same antiserum was 84 μm, the same neuron was not counted twice.

### Subcellular fractionation and Western blot analysis

Adult male mouse nucleus accumbens, dorsal striatum and cortical punches were sonicated and boiled in 1% SDS, 50 mM Tris [pH 7.4], 5 mM EDTA, 50 mM NaF, 2 mM sodium orthovanadate, 1 mM PMSF and protease inhibitor cocktail [17]. For subcellular fractionation studies, whole striatum was homogenized, fractionated by differential centrifugation followed by sucrose gradient centrifugation and extraction with TX-100, and subjected to Western blot analysis. Where indicated, blots were quantified using Gene Tools (Syngene, Frederick, MD) [12,17,33]. Gels were loaded with equal protein using a Bicinchoninic Acid Assay and bovine serum albumin standard to determine protein concentrations (Pierce, Rockford, IL). Commercially available mouse monoclonal antibodies were used: βIII tubulin (TUJ1; Covance), NR2B (clone N59/20; NeuroMab), Cdk5 (Santa Cruz Biotechnology) and Rac1 (Transduction Labs; also 23A8, Millipore).

## Results

### Kal7 is expressed in the major neuronal cell types in nucleus accumbens and dorsal striatum

**Figure 1:**
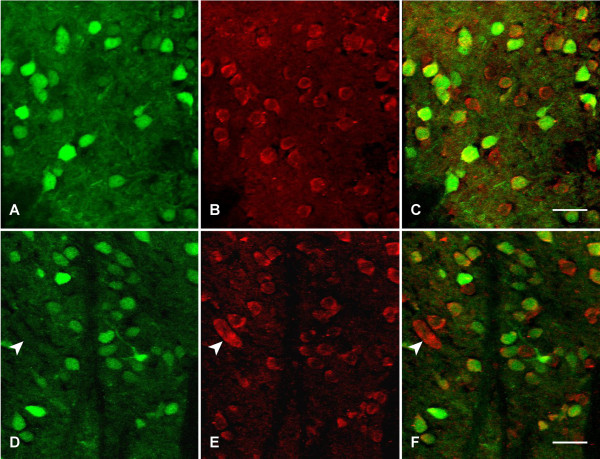
**Kal7 is expressed in the major neuronal cell types in nucleus accumbens and striatum**. Male Tg(Drd1a-EGFP)X60Gsat/Mmmh mice were deeply anesthetized, fixed by perfusion with 4% paraformaldehyde, and brains were sectioned as described [17]. Sections were stained for GFP (green) and Kal7 (red) and imaged with a confocal microscope. **A-C**. NAc core. **D-F**. Striatum. Scale bar, 20 μm. A collapsed Z-stack (0.5 μm steps) is shown. A total 249 neurons in six sections from two mice (GFP+Kal7, Kal7 only and GFP only) were counted in the striatum: 98% of the GFP-positive neurons contained Kal7 and 2% did not; 33% of the Kal7-positive neurons did not contain GFP. A total of 239 neurons in six sections from two mice were counted in the NAc: 92% of the GFP-positive neurons contained Kal7 and 8% did not; 31% of the Kal7-positive neurons did not contain GFP.

Understanding the role Kal7 plays in the effects of cocaine on locomotor activity and place preference requires knowing the striatal cell types in which it is expressed. Mice expressing GFP under the control of the D1 dopamine receptor (Drd1a-GFP mice) were used to investigate the cellular specificity of Kalirin expression in the dorsal striatum and nucleus accumbens (Figure 1). In both regions, more than half of the neurons express GFP, identifying them as D1 receptor positive medium spiny neurons; this ratio is consistent with published studies [34,35]. Over 90% of the D1 receptor neurons express Kal7. About one-third of the neurons in both regions express Kal7 but do not express GFP. D1 receptor negative neurons that are similar in size to the D1 receptor positive neurons represent medium spiny neurons expressing the D2 dopamine receptor [34,35]. The small population of very large neurons expressing high levels of Kal7 are aspiny cholinergic interneurons (Figure 1D-F) [36]. Kal7 could play a role at excitatory synapses onto each of these different neuronal cell types.

#### Usage of promoters encoding full-length mouse *Kalrn *is region-specific

**Figure 2:**
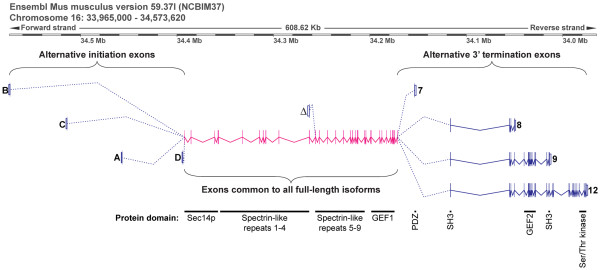
**Structure of the mouse *Kalrn *gene and major transcripts**. Genomic and transcript information was compiled from websites (http://www.ensembl.org/Mus_musculus/Info/;http://genome.ucsc.edu/cgi-bin/) and our previous work [26-28] to create an accurate map of the mouse *Kalrn *gene and the major brain transcripts, as described in Methods. Protein domains (http://smart.embl-heidelberg.de/) encoded by groups of exons are indicated.

With the generation of mice lacking the 3'-terminal exon unique to Kal7 and ΔKal7 (Kal7^KO ^mice) [17] and the distinctly different response of Kal7^KO ^mice to chronic cocaine exposure [12], a detailed analysis of *Kalrn *promoter usage and isoform expression in *Mus musculus *was undertaken (Figure 2). The genes encoding rat and human *Kalrn *are complex, with multiple initiation exons and multiple 3'-terminal exons responsible for the tissue-specific production of transcripts encoding functionally distinct proteins [26-28]. The gene encoding mouse *Kalrn *is similarly large, extending over 600 kb (Figure 2). Expression of full-length isoforms of *Kalrn*, in which a Sec14p domain and nine spectrin-like repeats precede the first GEF domain, is governed by four promoters and their alternative initiation exons (B, C, A, and D) located 1.4 to 180 kb upstream of exon 2 [27,28] (Figure 2). The alternative initiation exons, which are separated by large introns, encode unique N-termini that are five to 38 amino acids long. Exons 2 to 5 (35 kb) encode the common Sec14p-like domain while exons 5 through 22 (150 kb) encode the nine spectrin-like repeats. Exons 24 through 32 encode the first GEF domain. The 3'-terminal exon unique to Kal7 and ΔKal7 is flanked by two large introns while the exons encoding the second GEF domain (exons 39-47), which is present in Kal9 and Kal12, and the Ser/Thr kinase domain (exons 58-60), which is unique to Kal12, are densely clustered (Figure 2).

**Figure 3:**
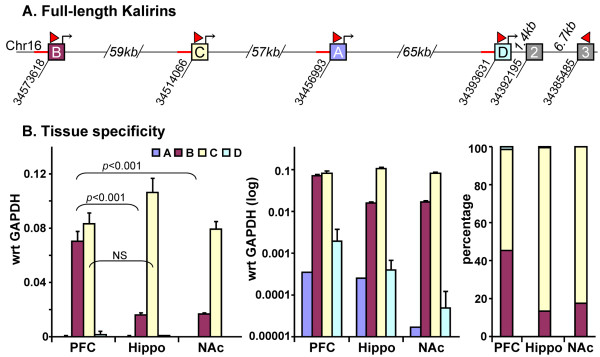
**Full-length Kalirin promoter usage varies in different brain regions**. **A**. Putative promoter regions (red line) and initiation exons in the mouse *Kalrn *gene, which is located on mouse chromosome 16, were identified based on homology to human and rat *Kalrn *[27,28]; translational start sites are indicated by bent arrows. The introns separating promoters B, C, A and D, which produce full-length Kalirin, are not drawn to scale. The location of each initiation exon is indicated by the nucleotide number below the line; the location of the initiation exon-specific forward primers is indicated, along with the common reverse primer in Exon 3. **B**. RNA prepared from adult mouse prefrontal cortex (PFC), hippocampus (Hippo) and nucleus accumbens (NAc) was subjected to Q-PCR analysis using these primers; data were normalized to GAPDH. The same data are plotted on a log scale to allow visualization of data for full-length Kalirin promoters A and D, and as a percentage of the total in each brain region.

Full-length Kalirin promoter usage was assessed by real-time Q-PCR; forward primers located in each initiation exon were paired with a common reverse primer located in exon 3 (Figure 3A). In addition to nucleus accumbens, RNA from prefrontal cortex and hippocampus was analyzed to look for region-specific promoter usage. Although PCR products of the expected size were detected for all four putative full-length promoters, *Kalrn *promoters B and C accounted for over 95% of the full-length Kalirin transcripts in the adult mouse brain (Figure 3B); usage of promoters A and D was detectable in all three brain regions, but was much less common. The promoter usage patterns for hippocampus and nucleus accumbens were similar to each other, but differed from the pattern observed in the prefrontal cortex. Full-length *Kalrn *promoters B and C were used equally in the prefrontal cortex, while promoter C was the major promoter in the hippocampus and nucleus accumbens.

#### Usage of Δ*Kalrn *promoter and alternate 3'-terminal exons is region-specific

**Figure 4:**
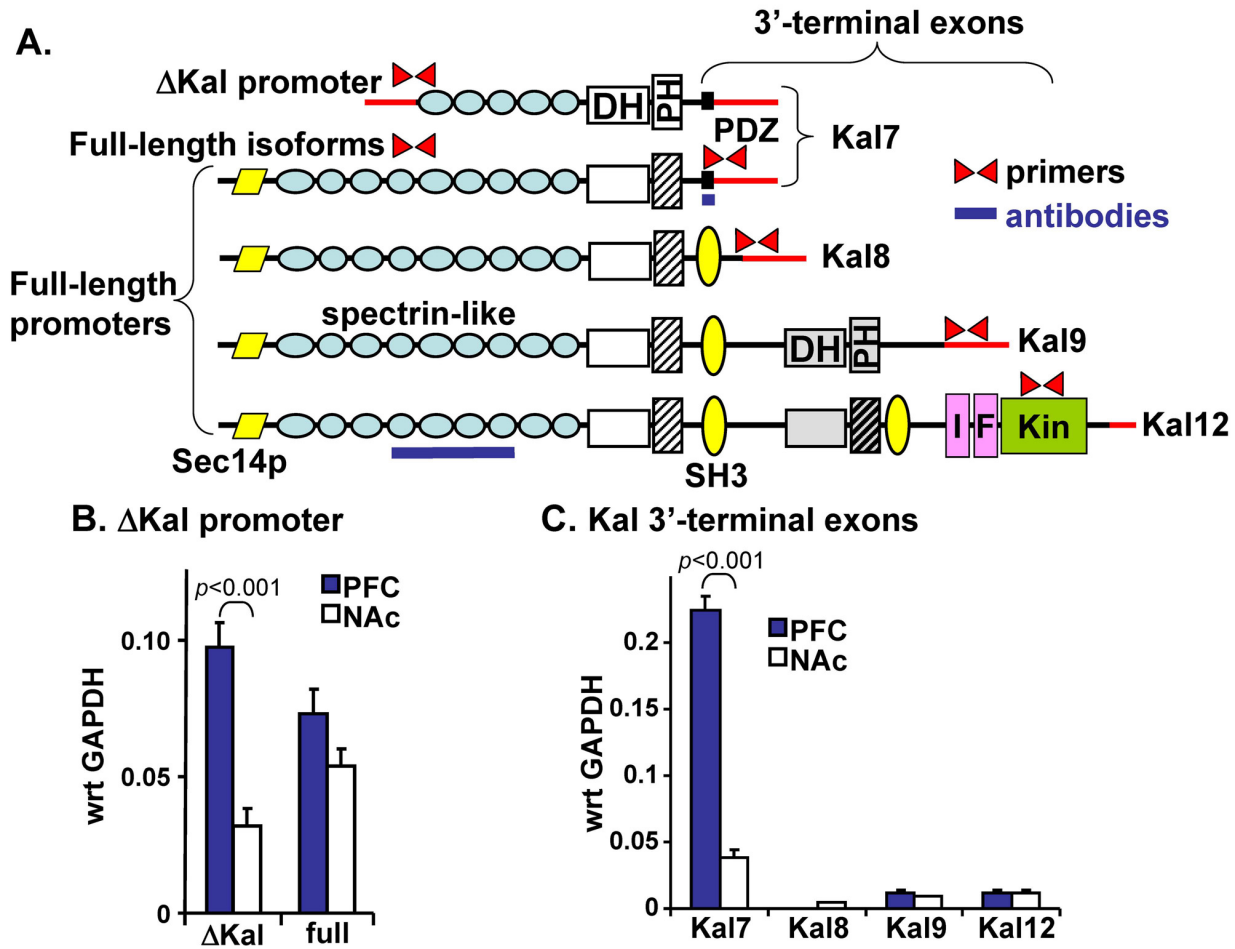
**Expression of transcripts encoding different Kalirin isoforms varies in nucleus accumbens and prefrontal cortex**. **A**. The proteins encoded by the major isoforms of rat and human Kalirin are drawn to scale; although Δ-isoforms of Kal8, -9 and -12 have been identified [26-28], only ΔKal7 is depicted. The ΔKal isoforms are produced when the ΔKal promoter is used instead of the A, B, C, or D promoters diagramed in **Figure 2**. The sense and anti-sense primers used for real-time Q-PCR are shown in red; 5'- and 3'-untranslated regions of the corresponding transcripts are indicated by red lines. The Kal7-specific and Kal-spectrin antibody epitopes are indicated by blue lines. **B**. RNA prepared from mouse nucleus accumbens (NAc) and prefrontal cortex (PFC) was subjected to real-time Q-PCR analysis using the ΔKal and full-length primers; three separate preparations of RNA from each tissue were analyzed in triplicate and data were normalized to GAPDH. **C**. PFC and NAc expression of Kalirin isoforms was examined via Q-PCR analysis of 3'-terminal exons encoding Kal7, Kal8, Kal9 and Kal12. Higher levels of Kal7 transcript were seen in the PFC than the NAc.

In the adult rat brain, usage of a promoter located in the intron that separates exons 10 and 11 yields the Δ-isoforms of Kalirin (Figures 2 and 4A). These isoforms lack the Sec14p domain and the first four spectrin-like repeats of full-length Kalirin. In rat cortical and hippocampal neurons, expression of exogenous Kal7 causes the formation of new dendritic spines. In contrast, expression of ΔKal7 increases spine size, but does not increase spine number [24].

Expression of transcripts encoding full-length vs. Δ-isoforms of Kalirin in mouse prefrontal cortex and nucleus accumbens was compared using Q-PCR with the indicated primers (Figure 4A); these regions were analyzed because both play essential roles in the response to cocaine and their patterns of promoter usage differed. Transcripts encoding the Δ-isoforms of Kalirin were more prevalent in prefrontal cortex than in nucleus accumbens (Figure 4B). Based on using primer pairs that spanned exons 10 and 11 (Figure 4A), transcripts encoding full-length isoforms of Kalirin were expressed at similar levels in these two brain regions.

Primers specific to the 3'-terminal exons unique to the different *Kalrn *isoforms (Figures 2, 4A) were used to look for differences between prefrontal cortex and nucleus accumbens (Figure 4C). Q-PCR analysis revealed much higher levels of transcripts encoding the Kal7-specific exon in prefrontal cortex than in nucleus accumbens. These transcripts could encode full-length Kal7 or ΔKal7. The Kal7-specific exon encodes a PDZ-binding domain known to interact with PSD-95 and spinophilin [18]. Transcripts encoding Kal8, Kal9 and Kal12 were detected at much lower levels in both tissues, with no tissue-specific differences. The isoforms of Kalirin that include both GEF1 and GEF2 (Kal9, Kal12) account for a larger fraction of the Kalirin transcripts in the nucleus accumbens than in the prefrontal cortex.

#### Kal7 is the major Kalirin protein in the dorsal striatum and nucleus accumbens

**Figure 5:**
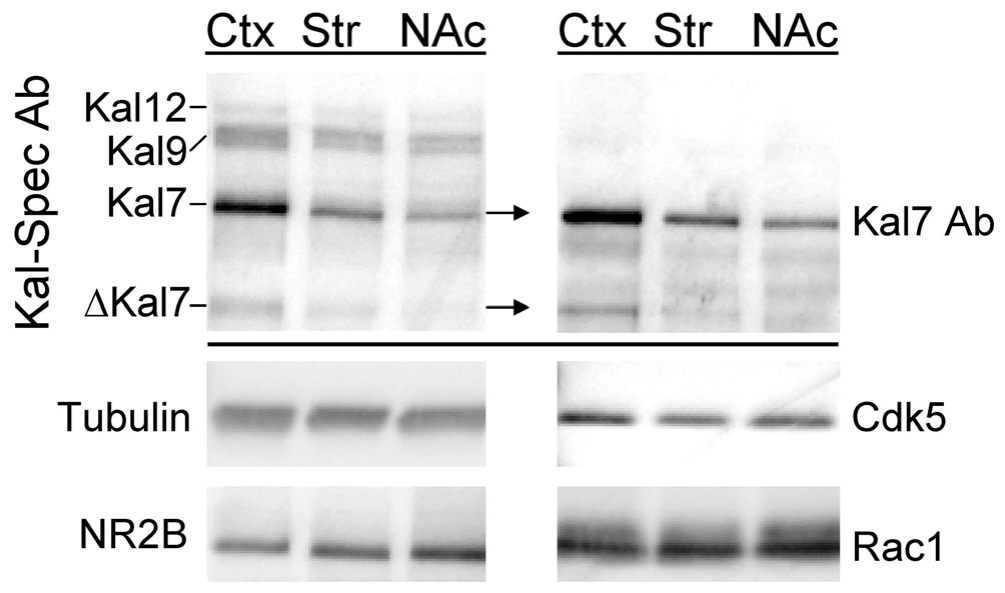
**Kalirin is not as highly expressed in the striatum as in the cortex**. **A**. Tissue taken from the cortex (Ctx), dorsal striatum (Str) and nucleus accumbens (NAc) of adult C57BL/6 mice was disrupted by sonication in SDS lysis buffer; aliquots containing equal amounts of protein (20 μg) were fractionated by SDS-PAGE and visualized using antibody to the spectrin repeat region of Kalirin or to Kal7 and the indicated proteins: βIII tubulin, NR2B, Cdk5 and Rac1.

Western blot analysis was used to compare expression of the major Kalirin isoforms in adult mouse cortex, where its role in spine formation has been studied [17,29,30,32,37-39] to dorsal striatum and nucleus accumbens (ventral striatum), the brain regions affected most dramatically by cocaine [12,35,40-47]. Using an antibody specific for spectrin repeats 4-7 of Kalirin (Kal-Spec Ab), cross-reactive proteins the size of Kal12 (337 kDa), Kal9 (271 kDa), Kal7 (190 kDa) and ΔKal7 (117 kDa) were detected (Figure 5, **top left**). Higher levels of Kal12, Kal7 and ΔKal7 were present in cortex than in dorsal or ventral striatum. In contrast, levels of βIII tubulin were similar in all three brain regions.

To confirm the identity of the bands identified as Kal7 and ΔKal7, antiserum specific to the unique COOH-terminus of Kal7 was used (Figure 5, **top right**). Levels of both Kal7 and ΔKal7 were higher in cortex than in striatum or nucleus accumbens, with a clear signal for ΔKal7 detected only in cortex. As observed in the rat [26,28], levels of ΔKal7 protein were significantly lower than predicted based on utilization of the Δ*Kalrn *promoter (Figure 4B).

Kalirin function is altered when Thr^1590 ^is phosphorylated by cyclin dependent kinase 5 (Cdk5), and levels of both Cdk5 and NR2B are decreased in PSDs prepared from Kal7^KO ^animals [17,30]. We therefore compared levels of NR2B and Cdk5 in these three brain regions (Figure 5); similar levels of both proteins were detected in all three regions. We also examined levels of Rac1, one of the major substrates of Kal7, in the same brain regions; again, no dramatic differences were observed (Figure 5).

#### Striatal PSDs are enriched in Kal7

**Figure 6:**
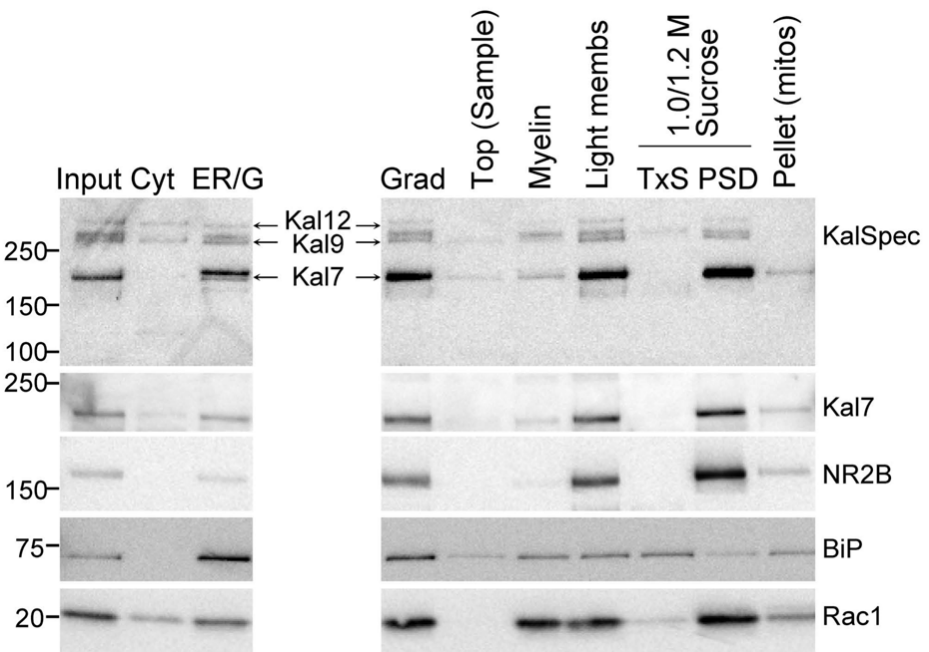
**Kal7 and Rac1 are enriched in mouse striatal PSDs**. Adult mouse striatum homogenized in buffer containing 0.32 M sucrose was subjected to differential centrifugation as described in Methods. After hypotonic lysis, synaptosomal membranes were pelleted, resuspended and subjected to equilibrium sucrose density centrifugation. Fractions were collected from the top (Sample) to bottom [Pellet (mitos)] of the gradient; the fraction taken from the interface of the 1.0/1.2 M sucrose layers was extracted with TX-100, yielding a TX-soluble fraction (TxS) and the PSD fraction. Equal amounts of protein (5 μg) from each fraction were subjected to SDS-PAGE; Kalirin was visualized using the Kal-spectrin antibody and the Kal7 antibody. The NR2B subunit of the NMDA receptor served as a PSD marker; BiP served as an endoplasmic reticulum marker. Rac1, one of the substrates of Kal-GEF1, was also localized to PSDs. Cyt = cytosol; ER/G = endoplasmic reticulum/Golgi-enriched fraction.

In the rat, mouse and gerbil cortex and hippocampus, subcellular fractionation reveals a significant enrichment of Kal7 at the PSD [3,17,18]. We prepared PSDs from mouse striatum to examine the distribution of the different Kalirin isoforms in this brain region (Figure 6). The distributions of BiP, an ER resident chaperone, and the NR2B subunit of the NMDA receptor, were used to verify the success of the fractionation scheme. As expected, BiP was enriched in the P3 fraction, which contains ER, Golgi and other intracellular membranous compartments, and NR2B was enriched in the PSD fraction. Cytosol was enriched in Kal12 and Kal9 and was devoid of Kal7. Kal7 was enriched in the PSD fraction, which also contained detectable amounts of Kal12 and Kal9, but no detectable ΔKal7.

Since Rac1 is one of the key substrates of Kal7, its distribution was also monitored (Figure 6). Although attached to the membrane by a geranylgeranyl tail, most of the Rac1 remained associated with the TX-100 washed PSD fraction. Very little Rac1 was removed by the TX-100 extraction step, leaving the TX-100 soluble fraction (TxS) depleted of Rac1.

**Figure 7:**
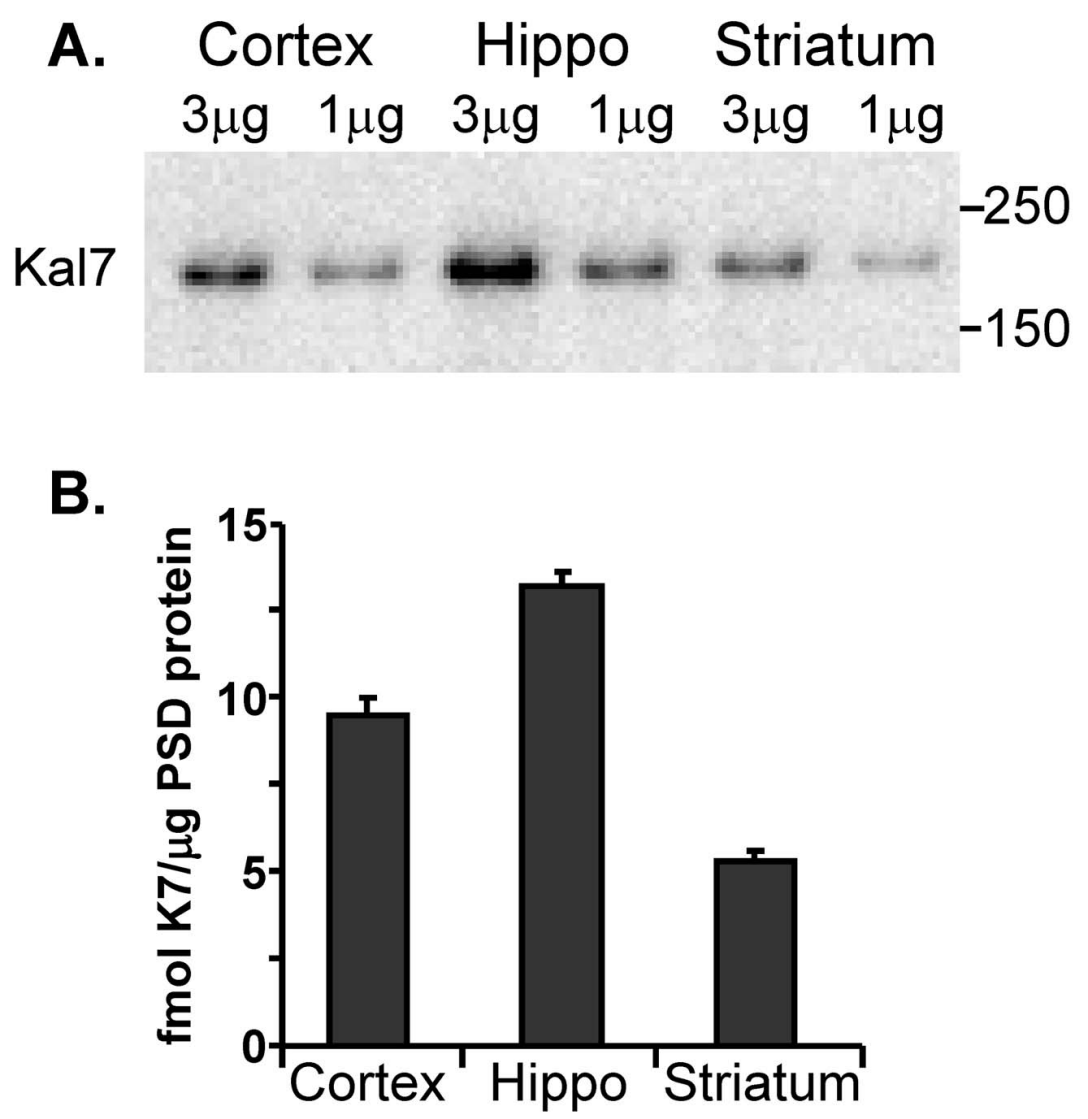
**Striatal PSDs contain less Kalirin than cortical or hippocampal PSDs**. TX-100 extracted PSDs were prepared from adult mouse cortex, hippocampus and striatum as described above. **A**. The indicated amount of protein was subjected to SDS-PAGE and Kal7 was visualized using the Kal7-specific antibody. **B**. Known amounts of purified ΔKal7 were analyzed at the same time, making it possible to calculate fmol Kal7/μg PSD protein using Gene Tools; data for two different amounts of protein were averaged; error bars show the range. The cortex and hippocampus numerical data in **B **are repeated from [17] for comparison.

We previously quantified the amount of Kal7 in purified PSDs prepared from adult mouse hippocampus and cortex [17]. Using purified ΔKal7 as a standard, the same measurement was made on purified striatal PSDs (Figure 7). The levels of Kal7 in striatal-PSDs were about half of those observed in the cortex and hippocampus. A difference of this magnitude is consistent with the results of *in situ *hybridization studies of Kalirin expression in these tissues [32].

### Chronic cocaine treatment alters promoter and 3'-terminal exon expression

**Figure 8:**
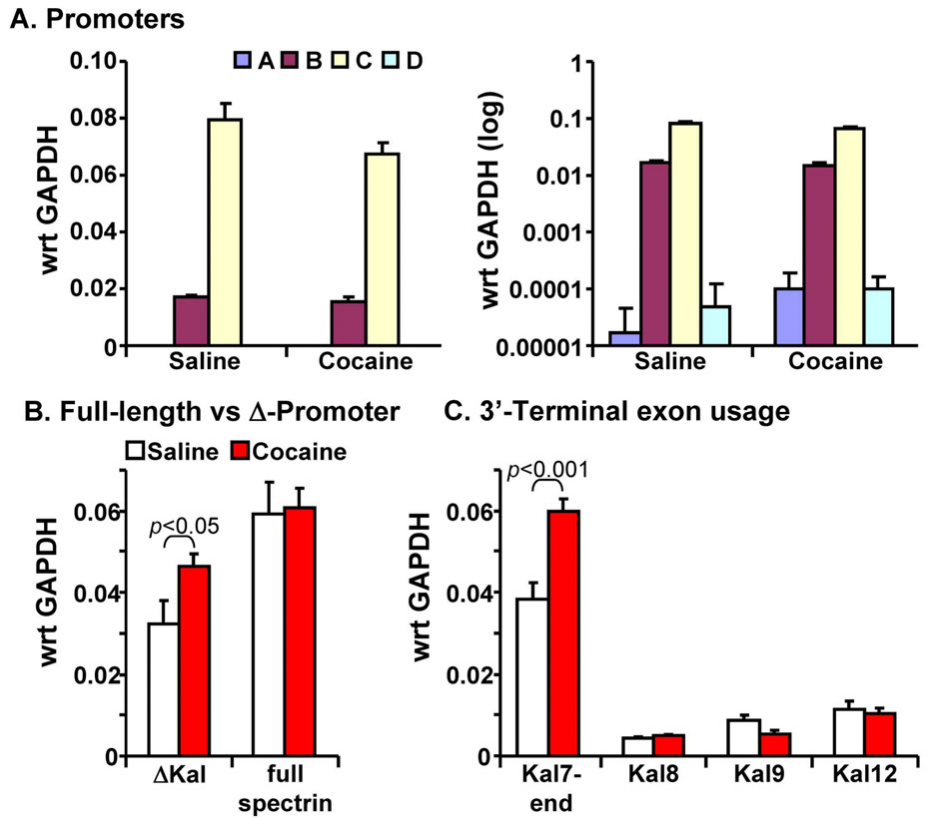
**Chronic exposure to cocaine increases usage of the ΔKal promoter and the Kal7 3'-terminal exon**. Samples of RNA were prepared from the nucleus accumbens of three sets of mice treated with cocaine (20 mg/kg for 7 days) or saline. **A**. Promoter usage was evaluated using the primers described in **Figure 1**. Cocaine treatment had no effect on usage of full-length *Kalrn *promoters A, B, C, or D. **B**. Chronic cocaine treatment increased usage of the ΔKal promoter. **C**. Usage of the different Kalirin 3'-terminal exons was evaluated as described in **Figures 2-3**; usage of the Kal7-specific 3'-terminal exon increased following chronic cocaine treatment.

Adult male mice were treated with cocaine (20 mg/kg) for 7 days and nucleus accumbens was then harvested for preparation of RNA (Figure 8). Utilization of full-length *Kalrn *promoters A, B, C and D was first evaluated; following injection of saline or cocaine, *Kalrn *promoter C accounted for most of the full-length transcripts produced (Figure 8A). Usage of the Δ*Kalrn *promoter was next compared to the total level of full-length *Kalrn *transcript (Figure 8B). Chronic cocaine treatment increased usage of the Δ*Kalrn *promoter without altering expression of full-length transcripts (Figure 8B).

The effect of chronic cocaine on usage of different *Kalrn *3'-terminal exons was next evaluated (Figure 8C). In the nucleus accumbens, the number of Kalirin transcripts generated using the Kal7-specific 3'-terminal exon increased following chronic treatment with cocaine. No significant change was seen in the level of transcripts encoding Kal8 or Kal12 and levels of Kal9 fell slightly (Figure 8C).

#### Effect of chronic cocaine on expression of Kalirin isoforms in striatal neurons

**Figure 9:**
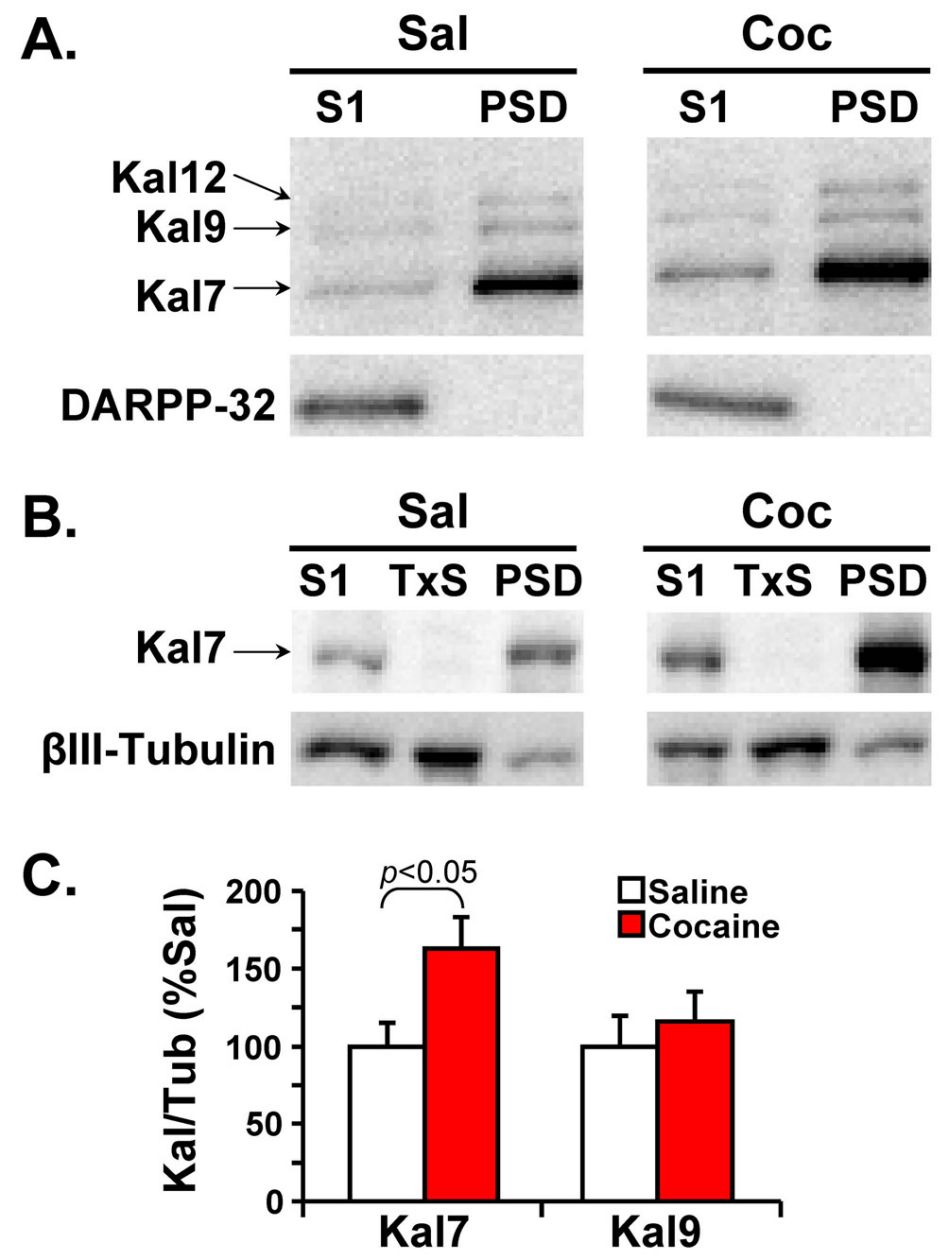
**Effect of chronic exposure to cocaine on expression of Kalirin protein**. **A**. Striata harvested from Saline and Cocaine treated mice were subjected to subcellular fractionation; equal amounts (10 μg) of input (S1) and PSD were fractionated by SDS-PAGE. Kalirin proteins were visualized using the Kal-spectrin antibody. DARPP32, a soluble protein highly expressed in the striatum, was excluded from the PSD fraction. **B**. Striata prepared in a similar fraction to A show increases in Kal7 in the S1 and PSD fractions while demonstrating a lack of Kal7 in the Triton soluble fraction as was expected from previous results [17,18]. **C**. Levels of Kal7 and Kal9 in SDS lysates prepared from the nucleus accumbens of chronically Saline and Cocaine treated mice were compared to levels of βIII-tubulin; Kal12 levels were not high enough to allow accurate quantification. The Kalirin/Tubulin ratios for Kal7 and Kal9 were calculated for Saline and Cocaine treated mice, with the Saline ratio normalized to 100%; using an unpaired t-Test, Kal7 expression (p < 0.05) differed significantly while Kal9 expression did not.

In order to evaluate changes in Kalirin isoform expression, PSDs were prepared from the striata of mice treated chronically with saline or cocaine (Figure 9A). Analysis of equal amounts of input (S1) and PSD protein revealed increased levels of Kal7 protein in both samples. DARPP32, a soluble protein enriched in striatal neurons, was excluded from the purified PSDs, attesting to their purity. Small amounts of Kal9 and Kal12 could be detected in the purified PSDs, but Kal12 was barely detectable in the input samples. The identification of the Kal7 band was verified with the Kal7-specific antibody (Figure 9B).

Many of the effects of cocaine involve the nucleus accumbens, or ventral striatum. Since preparation of purified PSDs from this relatively small region would require the use of multiple animals, we evaluated the effects of chronic cocaine on total lysates. Western blot analyses of nucleus accumbens extracts with the Kal-spectrin antibody revealed a 60% increase in Kal7 protein levels (Figure 9C); Kal9 levels were unchanged following cocaine treatment and Kal12 levels were too low for accurate quantification.

## Discussion and Conclusions

### Tissue-specific use of full-length *Kalrn *Promoters

The pattern of *Kalrn *promoter usage differs in NAc and prefrontal cortex; the C-promoter accounts for most of the full-length transcripts in the NAc while the B- and C-promoters contribute equally in the prefrontal cortex. The rat *Kalrn *A promoter was the first one identified and came from a cDNA library prepared from P21 rat hippocampus and cortex following electroconvulsive shock [11]. Subsequent studies demonstrated that the *Kalrn *A promoter is a minor contributor in the adult rat, mouse and human tissues examined [27,28]. The *Kalrn *D promoter is also a minor contributor in adult brain, but is the dominant *Kalrn *promoter in the pituitary (not shown). The Duet promoter, which produces transcripts that lack all of Kal7, is rarely used in the rat or human brain [28] and was not examined in this study. Interestingly, the intron sizes shown for mouse in Figure 1 are virtually identical to those reported for rat and human [28].

The N-terminal sequences encoded by the mouse and rat *Kalrn *B promoters are identical, as are the N-terminal sequences encoded by the mouse and rat *Kalrn *C promoters. Most of the full-length Kalirin isoforms in the NAc will begin with the more hydrophobic N-terminal sequence encoded by the *Kalrn *C promoter [48]. The functional consequences of this difference have not yet been explored, but the proximity of this short sequence to the Sec14p domain, which is known to bind phosphatidylinositol-3-phosphate [24], suggests that future studies might identify an important role for the N-terminus of Kalirin.

### Cocaine regulation of Δ*Kalrn *promoter and Kal7 3'-terminal exon usage

Chronic cocaine treatment increased the prevalence of transcripts generated using the Δ*Kalrn *promoter (Figure 8B). Neither the level of full-length Kalirin transcripts nor usage of the *Kalrn *B and C promoters (Figure 8A) was altered by this treatment. Chronic cocaine treatment is known to increase expression of several transcription factors, including CREB, ΔFosB, MEF2 and Sp1, in the NAc [45,47,49-51]. Our own analyses demonstrated an increase in transcripts encoding CREB, MEF2 and Sp1 in the NAc of mice treated with this particular regimen of cocaine [52].

**Figure 10:**
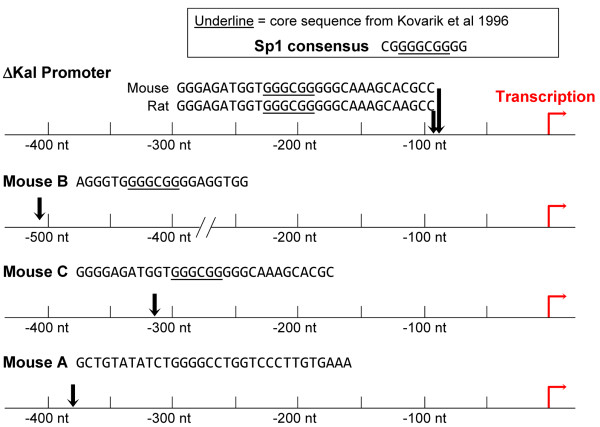
**Potential Sp1 sites in proximal regions of ΔKal, KalB and KalC promoters**. The 1 kilobase of genomic sequence 5' to the Δ, KalB and KalC transcriptional start sites was analyzed using Transcription Factor Search (http://www.cbrc.jp/research/db/TFSEARCH.html); mouse ΔKal (ENSMUST00000114949), rat ΔKal (AF229255.1), human ΔKal (BM920956.1; NM_001024660.2 starts 291 nt further 3'), mouse KalB (U88156.1) and mouse KalC (AF230644.1; NM_177357). The Sp1 consensus site definition is from [53]. Only one human cDNA corresponding to the mouse and rat ΔKal transcripts was identified; the 5' extent of the human transcript may be longer than depicted here, making the distance from the Sp1 site to the start of transcription shorter.

We therefore examined 1 kilobase of genomic sequence immediately upstream of the ΔKal, Kal B and Kal C transcriptional start sites for the presence of consensus binding sites for these four transcription factors (Figure 10). Consensus sites for CREB, ΔFosB and MEF2A were not found in these regions. A consensus Sp1 site (GGGCGG) was identified close to the Δ*Kalrn *transcriptional start site [53]; this site is perfectly conserved in mouse and rat. Consensus Sp1 sites, or close matches, were identified in the *Kalrn *B, *Kalrn *C and *Kalrn *A promoters, but are over 300 nucleotides from the transcriptional start site; none occur in the *Kalrn *D promoter (Figure 10). Because they are GC-rich, Sp1 sites are strongly affected by chromatin methylation [54]. Interfering pharmacologically with Sp1 function (e.g. with mithramycin) is known to block psychostimulant sensitization [51] and Sp1 binding to the proximal promoters of the mu opioid receptor has been clearly demonstrated [55].

Chronic treatment with cocaine also increased the level of transcripts that include the Kal7-specific 3'-terminal exon (Figure 8). Inclusion of this 3'-terminal exon results in the production of transcripts encoding Kal7 or ΔKal7 and precludes the production of Kal8, Kal9 or Kal12. Since chronic cocaine treatment had no effect on the level of full-length Kalirin transcripts (Figure 8B), an increase in the splicing events that include the Kal7-specific 3'-terminal exon would be associated with diminished usage of other 3'-terminal exons. While usage of the Kal8- and Kal12-specific exons was unaltered, usage of the Kal9-specific 3'-terminal exon declined slightly.

The effects of cocaine treatment on alternative splicing have received little attention. However, acute exposure to cocaine is known to alter splicing of transcripts encoded by the rat *BDNF *gene [56]. BDNF, acting through its TrkB receptor, plays an important role in the behavioral response to cocaine and enhances responsiveness to glutamate [57-59]. Cocaine- and activity-regulated alternative splicing of the Homer family of scaffolding proteins also affects synaptic signaling [43,60]. For *Kalrn*, splicing events that lead to the inclusion of different 3'-terminal exons produce proteins with distinctly different functions. Since the GEF2 region of Kalirin can be activated upon binding G_αq _[61], leading to activation of RhoA, even a slight shift in the ratio of isoforms with both GEF domains vs GEF1 alone should be of significance. Cocaine-induced alternative splicing of *Kalrn *transcripts may be important in the biochemical and/or behavioral response to cocaine.

### Kalirin expression in the striatum

The primary site of action of cocaine is the nucleus accumbens and the rest of the striatum [47,62,63]. Kal7 is expressed in almost all of the neurons in the dorsal striatum and nucleus accumbens that express the D1 receptor, but its expression is not limited to this population of neurons. Since medium spiny neurons expressing the D1 dopamine receptor and the D2 dopamine receptor receive different excitatory glutamatergic inputs and are part of very different functional pathways [34], examining the role of Kal7 in the effects of cocaine on both neuronal populations will be important. Whether expression of high levels of Kal7 in giant aspiny striatal cholinergic interneurons [36,63] plays an important role in the actions of cocaine will require additional studies.

Based on Q-PCR analysis, expression of Kalirin in the NAc is lower than in hippocampus or prefrontal cortex. Consistent with a role for Kalirin in spine formation, dendritic spine density on NAc medium spiny neurons is lower than on hippocampal CA1 pyramidal neurons [12,24]. In addition, PSDs purified from mouse striatum contained substantially lower levels of Kal7 than PSDs from cortex or hippocampus (Figure 7A). While the typical cortical or hippocampal PSD is estimated to have 9-12 molecules of Kal7, the typical striatal PSD would have only five.

Western blot analysis revealed an increase in levels of Kal7 in lysates of NAc and in purified striatal PSDs following chronic cocaine treatment (Figure 9). Many factors contribute to steady state protein levels, but the cocaine-stimulated increase in usage of the Kal7-specific 3'-terminal exon, coupled with maintained usage of the full-length *Kalrn *C promoter could account for the observed change. No cocaine-mediated alteration in Kal9 protein levels was observed (Figure 9B), despite a decrease in usage of the Kal9 specific 3'-terminal exon (Figure 8C). While ΔKal7 is detectable in mouse cortex, it is far less prevalent than predicted by promoter usage. The presence of four start codons followed by in-frame stop codons in the 5'-untranslated region of mouse ΔKal7 may limit successful translation [64], and increased turnover ΔKal7, a soluble, cytosolic protein, may contribute to this discrepancy.

A transient, cocaine-mediated change in levels of Kal7 vs. ΔKal7 would be expected to have functional significance. While expression of exogenous Kal7 increases spine density in cortical and hippocampal pyramidal neurons and even in interneurons [13,21,24], expression of exogenous ΔKal7 does not. Expression of ΔKal7 does increase spine head size [24] and the cocaine-mediated increase in Δ*Kalrn *promoter usage may contribute to the increase in spine width known to occur following cocaine administration [12,24]. Kal7 binding to its partners might be disrupted by ΔKal7, much as Homer-1a disrupts the clusters of proteins formed with the longer constitutive splice variants of the Homer family [60].

Initial studies on Kal7^KO ^mice confirmed a role for Kal7/ΔKal7 in nervous system function [12,17]. A slight decrease in linear spine density on CA1 hippocampal pyramidal neurons is accompanied by a decrease in spontaneous EPSP frequency and deficient hippocampal LTP [17]. In contrast, spine density is normal in the Kal7^KO ^NAc under basal conditions [12]. Although chronic administration of cocaine increases spine density on the medium spiny neurons in the NAc [35,42,65], this response is absent in Kal7^KO ^mice [12]. The behavioral responses of Kal7^KO ^mice to chronic cocaine are also altered [12]. Levels of both Cdk5 and NR2B, which play essential roles in the structural and behavioral responses to cocaine [17,30,41,66-69], are diminished in PSDs prepared from the cortices of Kal7^KO ^mice. A similar relationship between Kal7 and Cdk5 and NR2B in the NAc may contribute to the behavioral deficits observed.

Although the Na^+^-dependent plasma membrane dopamine transporter is the immediate target of cocaine, the effects of chronic exposure to cocaine are complex, wide-spread and long-lasting. Since no effective treatment is available, it is essential to develop a better understanding of the molecular, cellular and system-wide changes that lead to addiction. Structural changes at many of the synapses involved in addiction are thought to underlie these long-lived changes. Since dendritic spine morphology is largely controlled by the actin cytoskeleton, attention has turned to the pathways through which cocaine could alter the cytoskeleton. Small GTP binding proteins of the Rho family play an important role in this process and are activated by RhoGEFs and inactivated by Rho GTPase Activating Proteins. There are ~60 RhoGEFs in the human genome, and about a dozen are found in significant amounts at the PSD [16]. We have focused on one of these RhoGEFs, Kalirin, because it is essential for normal synaptic function and mice engineered to lack its major adult isoform exhibit altered responses to cocaine [12,17].

## Authors' contributions

REM and BAE conceived of the study, designed and performed the PCR analyses and westerns, and wrote the original draft of the manuscript. DDK designed and performed animal treatments and westerns. JEM designed and performed all bioinformatic analyses. XMM designed and performed the immunofluorescence studies. All authors participated in study design, read and approved the final manuscript.
